# Whole genome sequencing and comparative genomic analysis of oleaginous red yeast *Sporobolomyces pararoseus* NGR identifies candidate genes for biotechnological potential and ballistospores-shooting

**DOI:** 10.1186/s12864-020-6593-1

**Published:** 2020-02-24

**Authors:** Chun-Ji Li, Die Zhao, Bing-Xue Li, Ning Zhang, Jian-Yu Yan, Hong-Tao Zou

**Affiliations:** 1 College of Land and Environment, Shenyang Agricultural University, Shenyang, 110866 People’s Republic of China; 2 College of Agriculture and Biology, Zhongkai University of Agriculture and Engineering, Guangzhou, 510225 People’s Republic of China; 3 National Key Laboratory of Crop Genetic Improvement, Huazhong Agricultural University, Wuhan, 430070 People’s Republic of China; 4 College of Biological Science and Technology, Shenyang Agricultural University, Shenyang, 110866 People’s Republic of China

**Keywords:** *Sporobolomyces pararoseus*, Genome sequencing, Comparative genomic, Biotechnological potential, Ballistospores-shooting, Evolutionary direction

## Abstract

**Background:**

*Sporobolomyces pararoseus* is regarded as an oleaginous red yeast, which synthesizes numerous valuable compounds with wide industrial usages. This species hold biotechnological interests in biodiesel, food and cosmetics industries. Moreover, the ballistospores-shooting promotes the colonizing of *S. pararoseus* in most terrestrial and marine ecosystems. However, very little is known about the basic genomic features of *S. pararoseus*. To assess the biotechnological potential and ballistospores-shooting mechanism of *S. pararoseus* on genome-scale, the whole genome sequencing was performed by next-generation sequencing technology.

**Results:**

Here, we used Illumina Hiseq platform to firstly assemble *S. pararoseus* genome into 20.9 Mb containing 54 scaffolds and 5963 predicted genes with a N50 length of 2,038,020 bp and GC content of 47.59%. Genome completeness (BUSCO alignment: 95.4%) and RNA-seq analysis (expressed genes: 98.68%) indicated the high-quality features of the current genome. Through the annotation information of the genome, we screened many key genes involved in carotenoids, lipids, carbohydrate metabolism and signal transduction pathways. A phylogenetic assessment suggested that the evolutionary trajectory of the order Sporidiobolales species was evolved from genus *Sporobolomyces* to *Rhodotorula* through the mediator *Rhodosporidiobolus*. Compared to the lacking ballistospores *Rhodotorula toruloides* and *Saccharomyces cerevisiae*, we found genes enriched for spore germination and sugar metabolism. These genes might be responsible for the ballistospores-shooting in *S. pararoseus* NGR.

**Conclusion:**

These results greatly advance our understanding of *S. pararoseus* NGR in biotechnological potential and ballistospores-shooting, which help further research of genetic manipulation, metabolic engineering as well as its evolutionary direction.

## Background

Genomic studies of the oleaginous red yeasts have gained increased attention due to their great biotechnological potential for biomass-based biofuel production [1–4]. The red yeast *Sporobolomyces pararoseus* (previously known as *Sporidiobolus pararoseus*) belongs to the order Sporidiobolales [5], which is classified in the subphylum Pucciniomycotina, an earliest branching lineage of Basidiomycota. This species has been documented from a broad spectrum of environments, ranging from freshwater and marine ecosystem, soil, and to plant tissue [6]. Biomass of this yeast constitutes sources of carotenoid, lipid, exopolysaccharide, and enzyme [7, 8]. Colony color of *S. pararoseus* includes shades of pink and red due to the presence of lipid droplets full of carotenoid pigments, containing β-carotene, torulene and torularhodin [9–11].

However, there is little information on bioactivity and nutritional value of torulene and torularhodin, perhaps because they are rare in food, but its structure and sparse evidence provide some hints. For example, tests performed on human and mice showed that torulene and torularhodin have anti-prostate tumor activity [12]. Furthermore, torularhodin represents antimicrobial properties, and it may become a new natural antibiotic [13]. Previous studies have reported their safety to be used as a food additive [14]. In consideration of their valuable properties, torulene and torularhodin might be successfully used as food and pharmaceutical industries in the future. Members of the order Sporidiobolales comprise of genera *Sporobolomyces*, *Rhodosporidiobolus*, and *Rhodotorula*, are known as competent producers of torulene and torularhodin [15]. Consequently, genetic manipulation of *S. pararoseus* for large-scale torulene and torularhodin production will be one of the major aims of future research efforts.

Additionally, *S. pararoseus* is regarded as one of the most efficient microorganisms for bioconversion of crude glycerol into lipids [16]. Lipids content comprises from 20% up to 60% of the dry biomass [16]. These lipids are not only important sources of polyunsaturated fatty acids, such as arachidonic acid and docosahexaenoic acid, but also for the production of biodiesel [8]. Microbial lipids’ components are similar to that of vegetable oils, while have several advantages over vegetable oils [17, 18]. Such as a short life cycle, low space demands and independent of location and climates [19, 20]. Thus, the *S. pararoseus* also has been considered as potential feed stock for biodiesel industry [8].

Despite its long history of use for carotenoids fermentation, biodiesel production and ballistospores-shooting, very little is known about the basic genomic features of *S. pararoseus*. Advances in sequencing technology have drastically changed the strategies for studying genetic systems of microorganisms. Here, we present the first de novo genome assembly of *S. pararoseus*, as well as genes prediction and annotation. Subsequently, we performed a comparative analysis to investigate candidate orthologous and specific genes between *S. pararoseus*, *R. toruloides* and *S. cerevisiae*. The gene inventories provide vital insights into the genetic basis of *S. pararoseus* and facilitate the discovery of new genes applicable to the metabolic engineering of natural chemicals.

## Results

### Genome assembly and assessment

Here, the genome of oleaginous red yeast *S. pararoseus* NGR was sequenced using the Illumina Hiseq 2500 platform. A total of 8347 Mb raw data was generated from two DNA libraries: a pair-end library with an insert size of 500 bp (2631 Mb) and a mate-pair library with an insert size of 5 kb (5716 Mb). After, removing adapters, low-quality reads and ambiguous reads, we obtained 6073 Mb clean data (Q20 > 95%, Q30 > 90%) for genome assembly. For the genome size estimation of *S. pararoseus* NGR, we calculated the total 15 *k*-mer number is 705,505,006 and the *k*-mer depth is 28.41. According to the 15-mer depth frequency distribution formula, the estimated genome size of *S. pararoseus* NGR was calculated to be 24.44 Mb. Our final assembly consists of 54 scaffolds, a N50 length of 2,038,020 bp, the longest length scaffold of 4,025,647 bp, the shortest length scaffold of 513 bp, a GC content of 47.59% and a size of 20.9 Mb (85.52% of the estimated genome size). We identified 5963 genes in the genome with an average length of 1620 bp and a mean GC-content of 47.26% that occupied 55.07% of the genome. The results of BUSCO alignment showed that our final assembly contains 1273 complete BUSCOs (95.4%), of which 1268 were single-copy, while 5 were duplicated (Additional file 1). For the RNA-seq results, a total of 2662 Mb raw reads were generated. Using assessment of RNA-seq data, we found 98.68% (5884) of genes predicted in the NGR genome regions and 767 novel genes were expressed (Additional file 2). In addition, the RNA-seq data showed that 74.07% of reads matched to exon regions, 4.03% to intron regions, and 21.9% to intergenic regions. These reads are aligned to the intron region, mostly due to intron retention or alternative splicing events. In total 488 SNPs/InDel (Additional file 3) were identified when comparing RNA-seq data with the NGR genome sequences. From the RNA-seq data, we also identified the boundaries of 5’UTR and 3’UTR of 2772 genes (Additional file 4). Both BUSCO alignment and RNA-seq mapping suggested that our current genome assembly is characterized as high-quality, completeness and accuracy [21].

### Functional annotation

Among the 5963 predicted genes, 4595 (77.05%) genes could be annotated by BLASTN (E-value <1e^− 5^) using NCBI Nr databases based on sequence homology. In addition, 1940 (32.53%), 3002 (50.34%), 4237 (71.05%), 1806 (30.3%) and 4659 (78.13%) genes could be annotated according to KEGG, KOG, NOG, SwissProt, and TrEMBL databases, respectively. It should be noted that among these genes assigned to Nr database, the top 3 species of matched genes number are *R. toruloides* (3484, 75.82%), *Rhodotorula glutinis* (555, 12.08%) and *Microbotryum violaceum* (340, 7.4%). Furthermore, 4057 genes could be classified into three Gene Ontology (GO) categories (Additional file 5): cellular component (1883 genes), biological process (2802 genes), and molecular function (3388 genes). In addition, 194 tRNA, 1753 dispersed repetitive sequences, 2092 tandem repeats, 1178 minisatellite DNA (Additional file 6) and 659 micro-satellites DNA (Additional file 7) were identified in the genome. A total of 132,885 full-length TEs were predicted in the NGR whole genome. These TEs include 838 LTR-REs, 59 SINE-REs, 31 RC-REs, 598 DNA transposons, 208 LINE-REs and 7 Unknowns, of which 47.17% are Class LTR element, mainly assigned to Gypsy (346) and Copia (190). The full-length TEs totally comprised 132,885 bp, accounting for 0.61% of the NGR whole genome.

Based on KEGG pathways mapping, we annotated the coding genes of candidate for biotechnological potential in the NGR genome. A summary of the candidates (Additional files 8, 9, 10 and 11 for details) is presented as following: 1) carotenoids biosynthesis, including *crtI* (phytoene desaturase, GenBank: KR108014) [22], *crtYB* (lycopene cyclase/phytoene synthase, GenBank: KR108013) [23], *crtE* (GGPP synthase, GenBank: KY652916)*,* and other genes encoding hydroxylase, monooxygenase, or ketolase/carboxylase which might be responsible for the transformation from torulene to torularhodin; 2) lipid metabolism, including genes encoding acetyl-CoA carboxylase, acyl-CoA oxidase, phospholipid: diacylglycerol acyltransferase, glycerol 3-phosphate dehydrogenase; 3) carbohydrate metabolism, including genes encoding pyruvate dehydrogenase, pyruvate carboxylase and acyl-CoA: diacylglycerol acyltransferase; 4) stress responses, including genes involved in MAPK signaling pathway and calcium signal transduction.

### Phylogenetic relationships between red yeasts of the order Sporidiobolales

**Fig. 1:**
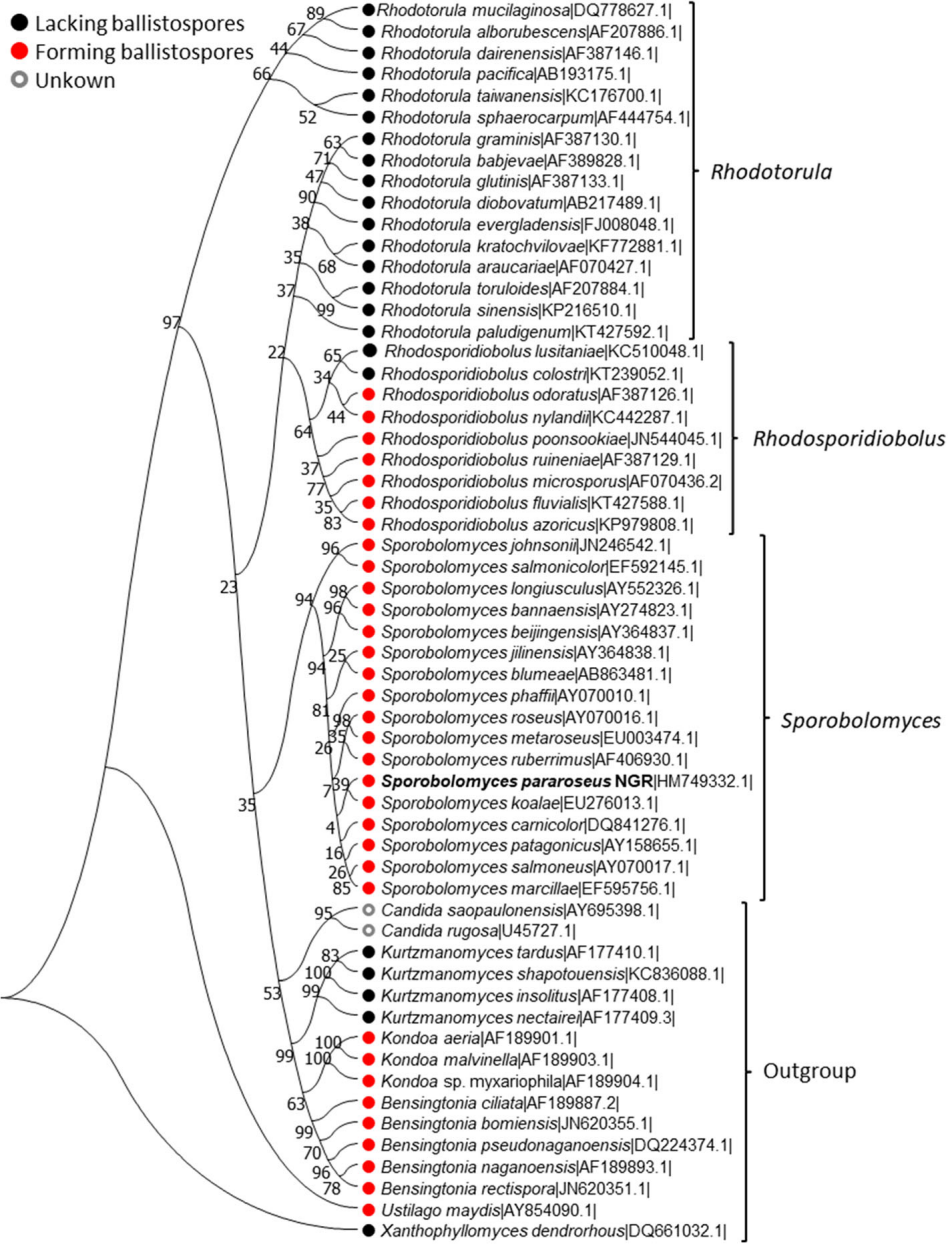
Phylogenetic tree of the order Sporidiobolales yeasts and outgroup species were constructed by Neighbor-Joining method and bootstrap analysis (1000 replicates) based on the alignment of the 26S rDNA sequence. The strain NGR font has been bolded. The numbers at the nodes indicate the bootstrap probabilities of the particular branch. Organisms belonging to the same genus have been represented on the right-side, representing as *Rhodotorula*, *Rhodosporidiobolus*, and *Sporobolomyces*. The scale (value: 0.01) representing nucleotide substitution per side is displayed. The accession numbers of the corresponding database entries are listed in behind the Latin name of each species. The ballistospores-forming ability for each entry of the phylogenetic tree is represented in front of the Latin name of each species. A red dot for those forming ballistospores, black dot for those not forming them and gray for those for which no information is available

Among phylum Basidiomycetes yeasts, there are a number of species that grow as pigmented colonies, and are for this reason known as red yeast [24]. Among them, 42 red yeasts belong to the order Sporidiobolales. Recently, the order Sporidiobolales has been reconstructed, including three genera *Sporobolomyces* (17 species) *Rhodosporidiobolus* (9 species) and *Rhodotorula* (16 species) [5, 25]. In order to determine the possible evolutionary trajectories between these red yeasts, we constructed the phylogenetic tree with available 26S rDNA sequences. As shown in Fig. 1, as for genus *Sporobolomyces*, the NGR showed a closer evolutionary relationship with *S. ruberrimus* and *S. koalae* than the other species, particularly for *S. johnsonii* and *S. salmonicolor*. The genus *Rhodosporidiobolus* situates a closer evolutionary relationship with *Rhodotorula* than with *Sporobolomyces*. The ballistospores are not uniform in the species of order Sporidiobolales, however, being a specialized mode of genus *Sporobolomyces* but absent in *Rhodotorula* and two characterized species of *Rhodosporidiobolus (R. lusitaniae and R. colostri)* [26–28]. It suggests that the same ancestor of *Sporobolomyces* and *Rhodosporidiobolus* species shoot ballistospores. However, the ballistospores-shooting ability was gradually lost in *R. lusitaniae/R. colostri* or other undescribed *Rhodosporidiobolus* species. Subsequently, some *Rhodosporidiobolus* species of lacking ballistospores-shooting ability has undergone a series of evolutionary processes to form *Rhodotorula* species. While these basic hypotheses are non-controversial, further verification basing on discovering more new Sporidiobolales species and obtaining their genome data is required.

### Comparative analysis of protein families and genes

**Fig. 2:**
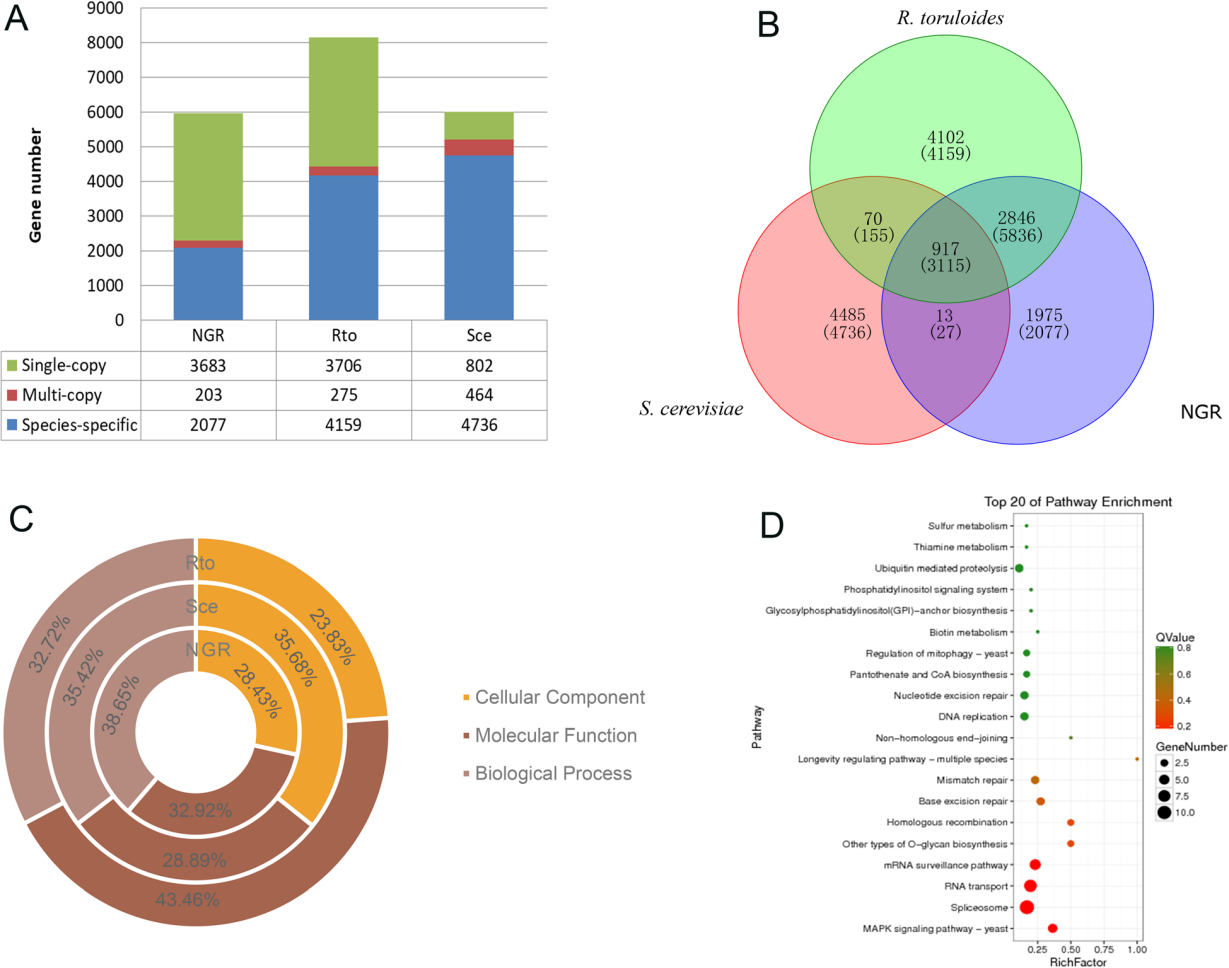
Comparative genomic analysis of *S. pararoseus* NGR, *R. toruloides* NP11, and *S. cerevisiae* S228C. **a** Distribution of single-copy, multi-copy and species-specific genes among three yeasts. **b** Venn diagram representation of shared/unique genes in *S. pararoseus* NGR and comparison with those in *R. toruloides* and *S. cerevisiae*. **c** Percentage of the gene numbers of species-specific protein families matched to different GO categories, in three yeast genomes, respectively. **d** Top 20 enriched KEGG pathways of species-specific genes in *S. pararoseus* NGR genomes. A rich factor is the ratio of the enriched genes numbers to total gene number in this pathway. The greater the rich factor, the higher the degree of enrichment. The Q-value ranges from 0 to 1 and the closer it is to zero, the more significant the enrichment

The NGR genome has predicted 5963 protein-coding genes, and the most of genes were annotated into the specie *R. toruloides* NP11. This motivates us to perform a comparative genomic analysis between *S. pararoseus* NGR and *R. toruloides* NP11. In order to exclude the inherent quality of yeast, we added the model yeast *S. cerevisiae* S288C as a control. As shown in Fig. 2a, we compared the distribution of genes among the three yeasts. In order to identify species-specific gene/protein families, we performed pairwise comparisons using a series of BLASTX searches within the three species. As shown in Fig. 2b, a total of 14,408 protein families were identified based on sequence similarities (5751 families for the NGR, 7935 families for NP11, and 5485 families for S288C)*.* 1975 (2077 genes), 4102 (4159 genes), and 4485 (4736 genes) protein families were species-specific in *S. pararoseus* NGR, *R. toruloides* NP11 and *S. cerevisiae* S288C, respectively. As shown in Fig. 2c, we conducted the GO analysis using respective species-specific genes of the three species. As for the genes of *S. pararoseus* NGR, 106 (16.4%), 280 (43.3%) and 261 (40.3%) terms were enriched in the CC, MF and BP, respectively. We found that the significantly enriched GO terms of the *S. pararoseus* NGR species-specific genes containing, CC: nucleus, membrane, and integral to membrane; MF: protein binding, DNA binding, and zinc ion binding; BP: regulation of transcription-DNA-dependent, transport, transmembrane transport, intracellular protein transport, carbohydrate metabolic process and oxidation-reduction process. Subsequently, we carried out the KEGG pathway mapping of *S. pararoseus* NGR species-specific genes. As shown in Fig. 2d, the significantly enriched pathways (Top 20) of the *S. pararoseus* NGR species-specific genes including MAPK signaling pathway-yeast, spliceosome, RNA transport, and mRNA surveillance pathways (Additional file 12).

Among the species-specific genes, NGR-1A3721 that assigned to the GO term of spore germination (GO: 0009847) was considered to be one of the candidates for the formation of ballistospores. Moreover, the species-specific genes of the NGR involved in the KEGG pathways of sugar metabolism, including amino sugar and nucleotide sugar metabolism (ko00520), pentose and glucuronate interconversions (ko00040), starch and sucrose metabolism (ko00500), galactose metabolism (ko00052), fructose and mannose metabolism (ko00051), and butanoate metabolism (ko00650) might be related to the ballistospores dissemination as reported in previous studies [29, 30]. Recently, Ianiri et al. reported that 3-hydroxyacyl-CoA dehydratase gene *Phs1* is not only responsible for the very long chain fatty acid biosynthesis, but also for the ballistospores-shooting in *Sporobolomyces* sp. IAM 13481 [31]. However, we found this *Phs1* gene in the both *S. pararoseus* NGR and *R. toruloides* genomes. Moreover, the *Phs1* gene was not strong positive or negative selected in substitution rates (Ka/Ks) analysis. Therefore, the *Phs1* should be an indirect determinant of the ballistospores-shooting in genus *Sporobolomyces*.

## Discussion

*S. pararoseus* is recognized as a kind of biotechnologically important oleaginous red yeast, which potentially can be used for biodiesel production as well as other important bio-products, such as carotenoids, enzymes and exopolysaccharide [32]. However, little is currently known about its genomic sequence and features. In the present study, the genome of *S. pararoseus* NGR will enable direct access to the genes responsible for its biology and biotechnological potential. To date, the only yeast belonging to the *Sporobolomyces* genus for which genome sequence is available is *S. salmonicolor* CBS 6832 [33]. As shown in Table 1, we compared the general genome features of *S. pararoseus* NGR and *S. salmonicolor* CBS 6832. The genome assembly quality of NGR is better than CBS 6832. The genome GC-content of CBS 6832 (61.3%) is higher than NGR (47.59%), but the predicted genes amount of CBS 6832 (5147) is less than NGR (5963). The *S. pararoseus* NGR genome will also serve as a useful basis of comparative genomics studies to investigate functional peculiarities specific to this yeast and its relative lineage within the *Sporobolomyces* clade.

**Table 1:** Genome features of *S. pararoseus* NGR and *S. salmonicolor* CBS 6832

Features	NGR	CBS 6832
Genome assembly size (Mb)	20.9	20.5
Number of contings	135	744
Number of scaffolds	54	395
Scaffolds N50 length (bp)	2,038,020	538,656
GC contents (%)	47.59	61.3%
Predicted genes (Nr)	5963	5147
Sequence platform	Illumina	Illumina + PacBio

**Fig. 3:**
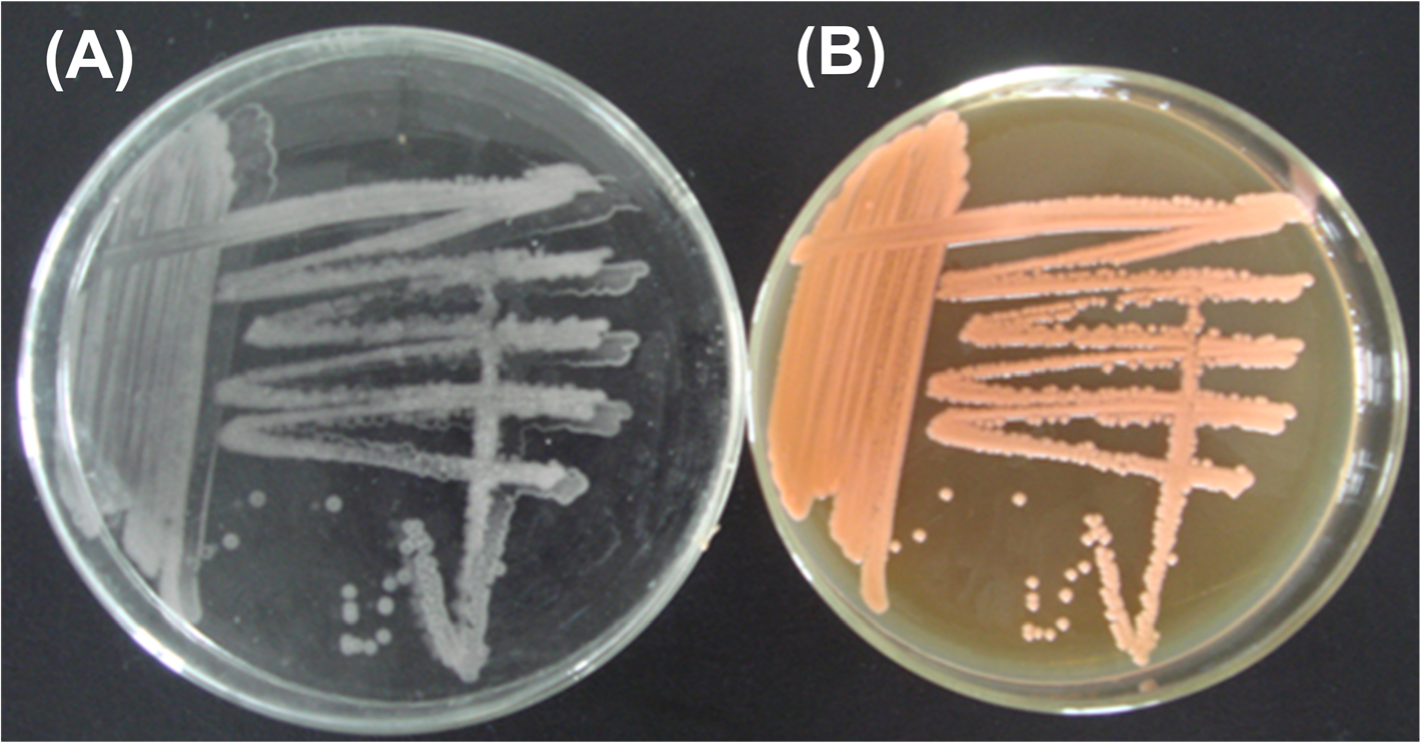
Ballistospores shoot in the *S. pararoseus* NGR. **a** The ballistospores have shot to the lid of the YPD plate to form mirror symmetry; **b** Colony morphology of the NGR patched on YPD plate. The trajectories of the ballistospores-shooting are perpendicular to the surface of the YPD plate. At the base of the ballistospores is a liquid droplet resulting from the drop coalescence that powers the explosive launch. The process of ballistospores-shooting is termed as “Buller’s drop” [29]

Moreover, one of the most notable characteristics of *S. pararoseus* is the process of ballistospores discharge. Ballistospores discharge is a unique type of spore produced by phylum Basidiomycetes fungi, however, does not occur in other fungal phyla [34]. As shown in Fig. 3, the *S. pararoseus* NGR was patched on agar medium to form colonies, and the ballistospores are vertically shot into the lid of the plate to form a “mirror” with their colonies. Ballistospores-booting is the main reason for this eukaryotic lineage colonizing in the most ecosystems. The *Sporobolomyces* species are endowed with many similar phenotypes with *Rhodotorula* species, such as carotenoids and lipid production, and morphological characteristics. However, an obvious difference between them is that *Rhodotorula* species are usually considered as marine microorganisms, and does not produce ballistospores. The ancestor of the order Sporidiobolales might be certain *Sporobolomyces* species and lived on land without the convenience of an aqueous environment. Dissemination of ballistospores is for finding new nutrient sources. As they entered and adapted to the marine environment, they gradually reduce the efficacy of ballistospores-shooting to form the *Rhodosporidiobolus* species and further lost ballistospores to evolve into the *Rhodotorula* species. Because of the ballistospores-shooting is widely considered as a biological process of energy consumption. When they exposed to excessive sea water, its energy should be preserved as much as possible to resist cold and high salt stresses, instead of discharging ballistospores. Both cold and salt stresses might play critical roles in positive selection and rapid evolution of genera *Sporobolomyces* to *Rhodotorula* species.

The Ka/ Ks ratio is widely considered to be an indicator of selective pressure during evolution [35]. To assess the overall difference in the selective restriction of gene levels within genera *Sporobolomyces* and *Rhodotorula*, the free ratio model was used to calculate the substitution rate for each orthologous gene [36]. Among the 700 pair’s single-copy homologous genes, we found that 80 pairs with a Ka/Ks value 0.1 < Ka/Ks < 0.5, 165 pairs with a Ka/Ks value Ka/Ks < 0.1, and 455 pairs with a Ks value = 0 (Additional file 13). The top four functional KEGG terms enriched among the negatively selected genes were “Carbohydrate metabolism”, “Translation”, “Lipid metabolism”, and “Amino acid metabolism”, which are associated with energy metabolism and the progress of protein synthesis or hydrolysis. This result indicates that the *Rhodotorula* species might have evolved a better energy metabolism and osmoregulation system to adapt to the marine environments and delay or prevent potential injury. But, the ballistospores-shooting is not necessary for its spreading in the marine environments. Given that the genes responsible for ballistospores-shooting still remain unknown. Our results provided valuable genetic data for the further characterization of the molecular mechanisms for ballistospores-shooting.

## Conclusions

Here, the high-quality *S. pararoseus* genome was reported. It established a genomic basis for further studying on its carotenoids, lipid, carbohydrate metabolism and stress responses. Furthermore, we proposed the evolutionary trajectories that *Rhodotorula* species were evolved from *Sporobolomyces* through the mediator *Rhodosporidiobolus.* Comparative genomic analysis revealed that the species-specific genes of *S. pararoseus* NGR related to spore germination and sugar metabolism, which might be involved in ballistospores-shooting. In conclusion, our work provides an important foundation for genes with potential biotechnological applications and foster comparative genomics studies to elucidate fundamental biological processes and evolutionary consequences of the order Sporidiobolales.

## Methods

### Strain material and DNA extraction

*S. pararoseus* NGR was isolated from strawberry fruit in the greenhouse of Shenyang Agricultural University (41°49′N, 123°34′E) in Shenyang City, Liaoning Province, China. Species identification was performed through morphological and molecular methods. The available GenBank accession number of *S. pararoseus* NGR 26S rDNA is HM749332. The strain number is recorded in the China General Microbiological Culture Collection Center as CGMCC 2.5280. *S. pararoseus* NGR cultures were grown for 72 h in 250 mL Erlenmeyer baffle flasks containing 50 mL of the YPD medium (10 g/L yeast extract, 20 g/L peptone and 20 g/L glucose, pH 6.5 ± 0.5) at 28 °C on a rotary shaker at 180 rpm. Genomic DNA of *S. pararoseus* NGR was extracted using the DNAiso Reagent kit (Code No.: 9770A) (Takara Bio, Dalian, China) according to the manufacturer’s protocols. The extracted genomic DNA was subjected to quality control by agarose gel electrophoresis and quantified by Qubit 2.0 fluorometer (Life Technologies, USA). The obtained genomic DNA (≥500 ng/μL) was used for whole genome sequencing and PCR verification.

### Genome sequencing

Genome sequencing of the strain NGR was performed utilizing the Illumina HiSeq 2500 platform (Illumina, USA). In order to obtain a high-quality de novo assembly, the strategy used was to combine data generated from standard short insert paired-end libraries with those from mate-pair libraries. Two DNA libraries were constructed: a paired-end library with an insert size of approximately 500 bp using TruSeq Nano DNA Kit (Illumina, USA) and a mate-pair library with an insert size of approximately 5 kb using Nextera DNA Library Preparation Kit (Illumina, USA). The 500 bp library and the 5 kb library were sequenced using the PE125 strategy at the Novogene Bioinformatics Technology Co., Ltd. (Beijing, China). After sequencing, quality control of the raw reads was performed, which involved trimming the reads using Trimmomatic (version 0.20) [37] by removing the Nextera adapter and linker sequences (for the mate-pair libraries) and TruSeq adapters (for the pair-end libraries); removing reads containing more than 10% of unknown nucleotides (N); removing low quality reads containing more than 50% of low quality (Q-value≤10) bases. For the trimmed reads, the online program FastQC (http://www.bioinformatics.babraham.ac.uk/projects/fastqc/) was used to plot quality score and sequence length distribution. Finally, the software ABySS (version 1.3.5) [38] was used to visualize the library complexity by plotting the *k*-mer profile of the reads. With these data, the genome size of the NGR was estimated by *k*-mer distribution (15 depth frequency) through the program KmerGenie (version 1.5621) with default parameters (inspired by FastQC) [39].

### Genome assembly

The filtered reads were assembled by SOAPdenovo2 (http://soap.genomics.org.cn/soapdenovo.html, version 2.0) under *k*-mer size of 15 [40–42] to generate scaffolds. The assembler SOAPdenovo2 follows the classic De Bruijn graph representation [43]. All reads were used for further gap closure using SOAPdenovo GapCloser Module as described in previous studies [42, 44]. Standard assembly statistics were obtained including: number of scaffolds, N50 (length N for which 50% of the entire assembly is contained in contigs or scaffolds equal to or larger than this value), N90 (same as N50 but using 90% instead), GC-content (%), the longest length scaffold, the shortest length scaffold, and total assembly length considering only scaffolds > 500 bp.

### Gene prediction and annotation

After obtaining the whole-genome sequence of the NGR, genes were predicted using the “Training module” and “Prediction module” of WebAUGUSTUS Service (http://bioinf.uni-greifswald.de/webaugustus/) with default parameters (strand = both; single strand = true; noInFrameStop = true) [45]. Repetitive sequences and distribution of transposable element (TEs) annotation were performed by using the program RepeatMasker (http://www.repeatmasker.org/, version v4.0.7) with default parameters based on libraries generated by different strategies: de novo-based, signature-based, and homology-based methods [46]. Tandem repeats were analyzed using the software Tandem Repeat Finder (http://tandem.bu.edu/trf/trf.html, version 4.09) with default parameters [47]. Ribosome RNA (rRNA) genes were predicted using the program rRNAmmer (http://www.cbs.dtu.dk/services/RNAmmer/, version 1.2) [48] with default parameters. Transfer RNA (tRNA) genes were predicted using the program tRNAscan-SE (http://lowelab.ucsc.edu/tRNAscan-SE/, version 2.0.4) with default parameters [49]. Non-coding RNAs were predicted by BLAST against Rfam (RNA families) database (http://rfam.xfam.org/, version 13.0) with default parameters [50, 51]. Functional annotation of the predicted genes was performed by similarity using BLAST against diverse public databases, including: the NCBI’s non-redundant protein (Nr) database, UniProt/Swiss-Prot, Cluster of Orthologous Groups of proteins (COG), Kyoto Encyclopedia of Genes and Genomes (KEGG) and Protein Families (Pfam) [52]. BLASTN searches were carried out using an E-value less than 1e^− 5^, minimal alignment length percentage larger than 40% (identity ≥40%, coverage ≥40%). The Gene Ontology (GO) annotations was performed using the program Blast2GO with default parameters [53] and GO-term classification was conducted based on the Nr annotations.

### Identification of orthologous genes

Annotations of coding sequences and proteins of *Rhodotorula toruloides* NP11 (https://www.ncbi.nlm.nih.gov/assembly/GCF_000320785.1/) and *Saccharomyces cerevisiae* S288C (https://www.ncbi.nlm.nih.gov/assembly/GCF_000146045.2/) were downloaded from NCBI Assembly database. The NGR, NP11 and S288C genome sequence alignments were performed in an all-against-all comparison using the MUMmer 3 package (http://mummer.sourceforge.net/, version 3.2.2) with default parameters [54]. Comparative genome analyses were performed at protein level. The software OrthoMCL (https://orthomcl.org/orthomcl/, version 2.0) was used to generate core-orthologs for the NGR, NP11 and S288C whole proteomes datasets with default parameters [55]. Subsequently, all the putative proteins of the three yeast species and core-orthologs were aligned (all against all) using BLASTP (http://www.ncbi.nih.gov/BLAST/) and a score for each pair of proteins with significant matches was assigned with a cut-off value of 1 × 10^− 7^ [56]. These species-specific genes of the NGR were used for screening the candidates for the formation and dissemination of ballistospores. Gene ontology (GO) and Kyoto Encyclopedia of Genes and Genomes (KEGG) enrichment analyses were performed using DAVID functional annotation tool (https://david.ncifcrf.gov/tools.jsp, version 6.8) with default parameters [57, 58]. Subsequently, we used the GO/KEGG enrichment results to further screen the candidate genes for ballistospores-shooting of the NGR.

### RNA-seq and gene models prediction

RNA was extracted from the NGR cells using Trizol Reagent Kit (Invitrogen, USA) and then checked by 1% agarose gel electrophoresis and a NanoPhotometer spectrophotometer (IMPLEN, CA, USA). The complementary DNA (cDNA) libraries were constructed using NEB Next Ultra RNA Library Prep Kit for Illumina (NEB, USA) following manufacturer’s recommendations. The constructed products were purified and then amplified with PCR to obtain the high-quality cDNA library and sequenced at an Illumina Hiseq 2000 platform using the SE100 strategy at the Novogene Bioinformatics Technology Co., Ltd. (Beijing, China). Raw reads of fastq format were firstly processed through the fastp program (version 0.18.0) (https://github.com/OpenGene/fastp) with default parameters [59]. Clean reads were obtained by the fastp program with following parameters: 1) removing reads containing adapters; 2) removing reads containing more than 10% of unknown nucleotides (N); 3) removing low quality reads containing more than 50% of low quality bases [59]. Q20, Q30, GC-content and sequence duplication level of the clean reads were calculated to assess the quality of clean reads. All the downstream analyses were based on clean reads with high quality.

These high-quality clean reads were first mapped to the NGR genome using the software HISAT2 (version 2.1.0) with “-rna-strandness RF” and other parameters set as a default [60, 61]. The mapped reads were assembled to reconstruct transcripts by using StringTie (version 1.3.1) in a “reference-based” approach [61, 62]. The reconstructed transcripts that are not annotated in the NGR genome are defined as novel genes [62]. Gene expression was then measured in fragments per kilobase of exon per million fragments mapped (FPKM) using the program StringTie (version 1.3.1) with default parameters [62]. After mapping reads to the NGR genome, the software HISAT2 (version 2.1.0) was used in reconstruction of transcripts which may extend the 5′ untranslated region (5′ UTR) or 3′ untranslated region (3′ UTR) of gene to optimize the gene structure. Calling variants of transcripts were carried out using the Genome Analysis Toolkit (GATK, version 4.1.4.1) with default parameters [63]. The software ANNOVAR (by default parameters) was used for single nucleotide polymorphisms (SNPs) and insertion-deletion (InDel) annotation [64].

### Assessment of genome completeness

Both orthologous gene alignment and RNA-seq data mapping were employed to evaluate our genome completeness. The program BUSCO (version 3.0.1) was applied to align the orthologs of the NGR to a reference gene set of basidiomycota_odb9 with default parameters [65].

### Substitution rate estimation and selection analyses

The substitution rates (Ka/Ks, the ratio of nonsynonymous to synonymous substitutions) for each orthologous gene were used to evaluate the overall differences in the selective pressure at the gene level within the three yeast lineages, using the free ratio model in KaKs_Calculator Toolbox (version 2.0) software with default parameters [66, 67]. The genes with *p*-value< 0.05 and a higher Ka/Ks value (Ka/Ks > 1) were considered to be the positively selected genes, as described in a previous study [68].

### Phylogenetic analysis

All 26S rDNA nucleotide sequences for the phylogenetic analyses were from NCBI Nucleotide database (https://www.ncbi.nlm.nih.gov/nuccore). All sequences were processed with the software MEGA (version 7.0) using Muscle alignment with UPGMB clustering method [69, 70]. Subsequently, all sequences were trimmed manually to remove the unaligned sequences of 3′ and 5′ end. The phylogeny was tested by applying Bootstrap method [71], Bootstrap values expressed as percentages of 1000 replications, are given at the branching points. Phylogenetic tree was constructed using these trimmed sequences by the Neighbor-Joining method. All analysis was carried out using default parameters without bootstrapping in the software MEGA 7.0.

## Supplementary information

**Additional file 1.** Genome completeness analysis through applied BUSCO software (version 3.0.1) to align the orthologs of the NGR to a reference gene set of basidiomycota_odb9. (XLS 71 kb)

**Additional file 2.** All genes expression profile and functional annotations resulted from RNA-seq analysis. (XLS 1904 kb)

**Additional file 3.** SNP/InDel annotations resulted from RNA-seq data. (XLS 48 kb)

**Additional file 4.** Gene structure optimization resulted from RNA-seq data.

**Additional file 5.** GO categories of 4057 genes resulted from genome analysis.

**Additional file 6.** Mini-satellite DNA annotation file of NGR.

**Additional file 7.** Micro-satellites DNA annotation file of NGR.

**Additional file 8.** All gene annotation information resulted from genome analysis.

**Additional file 9.** Genome annotations file of NGR.

**Additional file 10.** CDS/cDNA sequences resulted from genome analysis.

**Additional file 11.** Protein sequences resulted from genome analysis.

**Additional file 12.** GO/KEGG enrichment results of species-specific genes of the NGR.

**Additional file 13.** Ka/Ks results of single-copy homologous gene (NGR vs NP11).

## Data Availability

All the raw sequence data are available via GenBank under the SRA accessions SRX3638123-SRX3638125 (three raw data for the transcriptome) and SRR9733737- SRR9733739 (six raw data for the whole genome). This *S. pararoseus* strain NGR Whole Genome Shotgun project has been deposited at DDBJ/ ENA/ GenBank under the accession RSDY00000000; BioProject: PRJNA505991; BioSample: SAMN10440612. The version described in this article is version RSDY01000000.

## Abbreviations

BP: Biological process
CC: Cellular component
COG: Cluster of Orthologous Groups of proteins
GO: Gene Ontology
KEGG: Kyoto Encyclopedia of Genes and Genomes
MF: Molecular function
Nr: NCBI’s non-redundant protein database
Pfam: Protein Families
Rfam: RNA families
